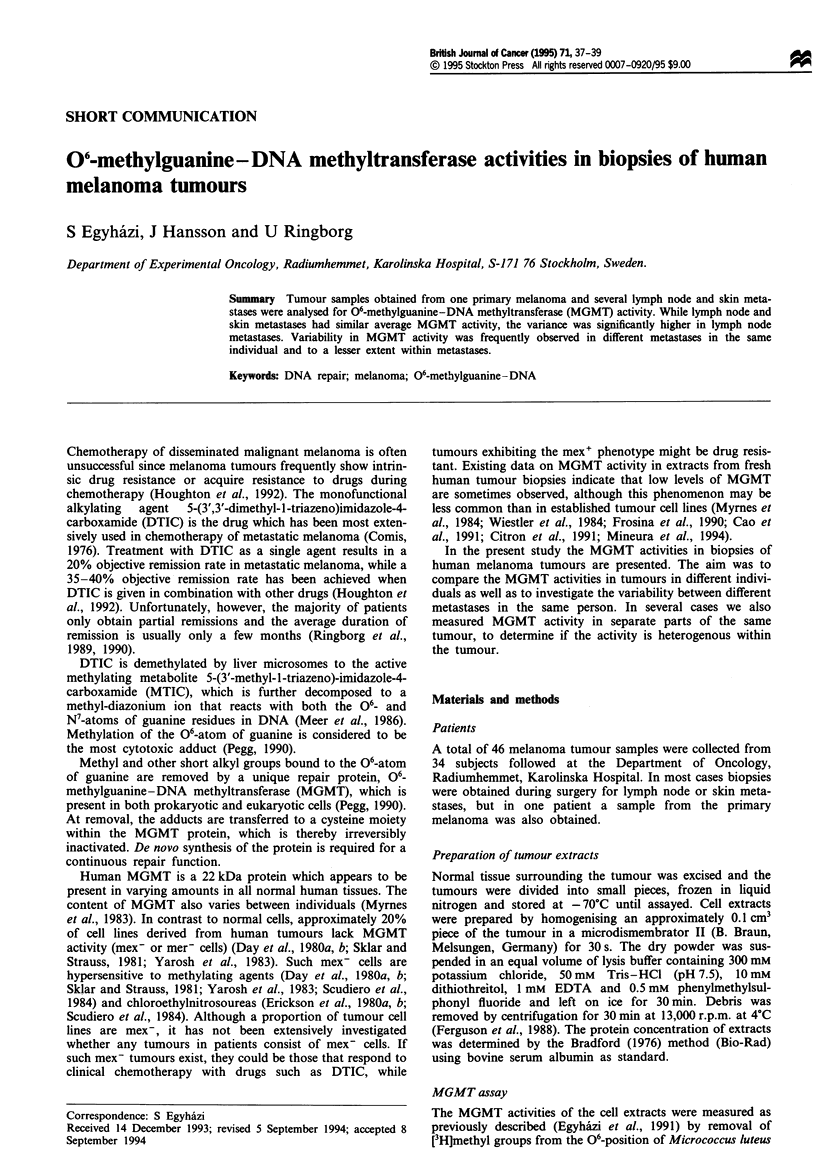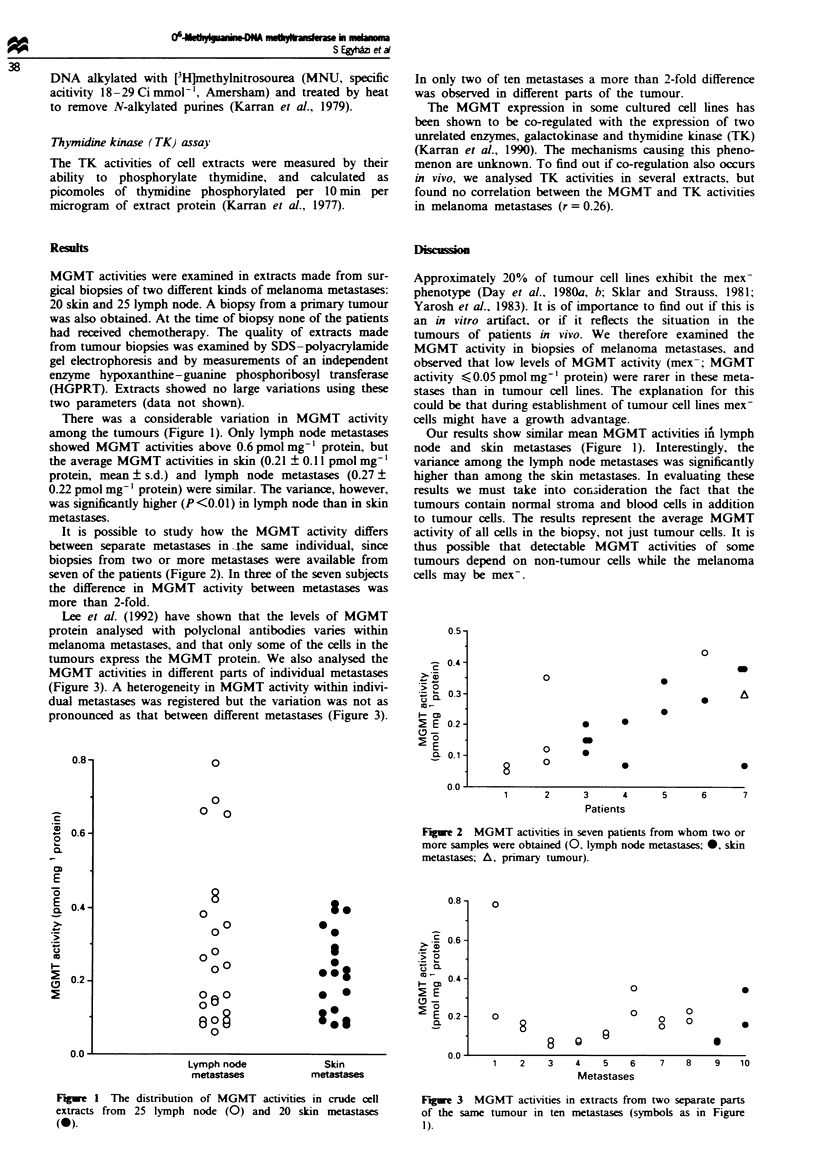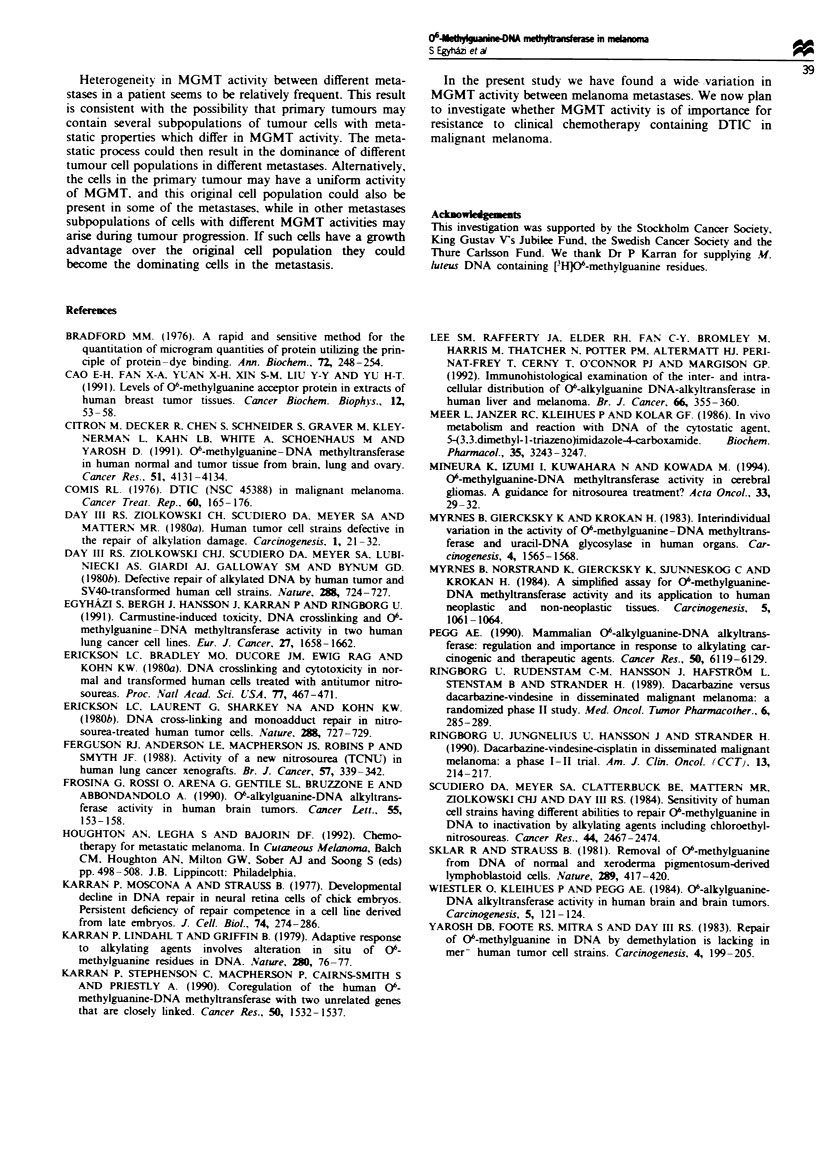# O6-methylguanine-DNA methyltransferase activities in biopsies of human melanoma tumours.

**DOI:** 10.1038/bjc.1995.8

**Published:** 1995-01

**Authors:** S. Egyházi, J. Hansson, U. Ringborg

**Affiliations:** Department of Experimental Oncology, Radiumhemmet, Karolinska Hospital, Stockholm, Sweden.

## Abstract

Tumour samples obtained from one primary melanoma and several lymph node and skin metastases were analysed for O6-methylguanine-DNA methyltransferase (MGMT) activity. While lymph node and skin metastases had similar average MGMT activity, the variance was significantly higher in lymph node metastases. Variability in MGMT activity was frequently observed in different metastases in the same individual and to a lesser extent within metastases.


© 1995 Stockton Press All rights reserved 0007–0920/95 $9.00

SHORT COMMUNICATION

### $O^6$-methylguanine–DNA methyltransferase activities in biopsies of human melanoma tumours

S Egyházi, J Hansson and U Ringborg

*Department of Experimental Oncology, Radiumhemmet, Karolinska Hospital, S-171 76 Stockholm, Sweden.*

**Summary** Tumour samples obtained from one primary melanoma and several lymph node and skin metastases were analysed for $O^6$-methylguanine–DNA methyltransferase (MGMT) activity. While lymph node and skin metastases had similar average MGMT activity, the variance was significantly higher in lymph node metastases. Variability in MGMT activity was frequently observed in different metastases in the same individual and to a lesser extent within metastases.

**Keywords:** DNA repair; melanoma; $O^6$-methylguanine–DNA

Chemotherapy of disseminated malignant melanoma is often unsuccessful since melanoma tumours frequently show intrinsic drug resistance or acquire resistance to drugs during chemotherapy (Houghton *et al.*, 1992). The monofunctional alkylating agent 5-(3′,3′-dimethyl-1-triazeno)imidazole-4-carboxamide (DTIC) is the drug which has been most extensively used in chemotherapy of metastatic melanoma (Comis, 1976). Treatment with DTIC as a single agent results in a 20% objective remission rate in metastatic melanoma, while a 35–40% objective remission rate has been achieved when DTIC is given in combination with other drugs (Houghton *et al.*, 1992). Unfortunately, however, the majority of patients only obtain partial remissions and the average duration of remission is usually only a few months (Ringborg *et al.*, 1989, 1990).

DTIC is demethylated by liver microsomes to the active methylating metabolite 5-(3′-methyl-1-triazeno)-imidazole-4-carboxamide (MTIC), which is further decomposed to a methyl-diazonium ion that reacts with both the $O^6$- and $N^7$-atoms of guanine residues in DNA (Meer *et al.*, 1986). Methylation of the $O^6$-atom of guanine is considered to be the most cytotoxic adduct (Pegg, 1990).

Methyl and other short alkyl groups bound to the $O^6$-atom of guanine are removed by a unique repair protein, $O^6$-methylguanine–DNA methyltransferase (MGMT), which is present in both prokaryotic and eukaryotic cells (Pegg, 1990). At removal, the adducts are transferred to a cysteine moiety within the MGMT protein, which is thereby irreversibly inactivated. *De novo* synthesis of the protein is required for a continuous repair function.

Human MGMT is a 22 kDa protein which appears to be present in varying amounts in all normal human tissues. The content of MGMT also varies between individuals (Myrnes *et al.*, 1983). In contrast to normal cells, approximately 20% of cell lines derived from human tumours lack MGMT activity ($mex^-$ or $mer^-$ cells) (Day *et al.*, 1980*a*, *b*; Sklar and Strauss, 1981; Yarosh *et al.*, 1983). Such $mex^-$ cells are hypersensitive to methylating agents (Day *et al.*, 1980*a*, *b*; Sklar and Strauss, 1981; Yarosh *et al.*, 1983; Scudiero *et al.*, 1984) and chloroethylnitrosoureas (Erickson *et al.*, 1980*a*, *b*; Scudiero *et al.*, 1984). Although a proportion of tumour cell lines are $mex^-$, it has not been extensively investigated whether any tumours in patients consist of $mex^-$ cells. If such $mex^-$ tumours exist, they could be those that respond to clinical chemotherapy with drugs such as DTIC, while tumours exhibiting the $mex^+$ phenotype might be drug resistant. Existing data on MGMT activity in extracts from fresh human tumour biopsies indicate that low levels of MGMT are sometimes observed, although this phenomenon may be less common than in established tumour cell lines (Myrnes *et al.*, 1984; Wiestler *et al.*, 1984; Frosina *et al.*, 1990; Cao *et al.*, 1991; Citron *et al.*, 1991; Mineura *et al.*, 1994).

In the present study the MGMT activities in biopsies of human melanoma tumours are presented. The aim was to compare the MGMT activities in tumours in different individuals as well as to investigate the variability between different metastases in the same person. In several cases we also measured MGMT activity in separate parts of the same tumour, to determine if the activity is heterogenous within the tumour.

### Materials and methods

### *Patients*

A total of 46 melanoma tumour samples were collected from 34 subjects followed at the Department of Oncology, Radiumhemmet, Karolinska Hospital. In most cases biopsies were obtained during surgery for lymph node or skin metastases, but in one patient a sample from the primary melanoma was also obtained.

### *Preparation of tumour extracts*

Normal tissue surrounding the tumour was excised and the tumours were divided into small pieces, frozen in liquid nitrogen and stored at −70°C until assayed. Cell extracts were prepared by homogenising an approximately 0.1 $cm^3$ piece of the tumour in a microdismembrator II (B. Braun, Melsungen, Germany) for 30 s. The dry powder was suspended in an equal volume of lysis buffer containing 300 mM potassium chloride, 50 mM Tris–HCl (pH 7.5), 10 mM dithiothreitol, 1 mM EDTA and 0.5 mM phenylmethylsulphonyl fluoride and left on ice for 30 min. Debris was removed by centrifugation for 30 min at 13,000 r.p.m. at 4°C (Ferguson *et al.*, 1988). The protein concentration of extracts was determined by the Bradford (1976) method (Bio-Rad) using bovine serum albumin as standard.

### *MGMT assay*

The MGMT activities of the cell extracts were measured as previously described (Egyházi *et al.*, 1991) by removal of [$^3$H]methyl groups from the $O^6$-position of *Micrococcus luteus*

Correspondence: S Egyházi
Received 14 December 1993; revised 5 September 1994; accepted 8 September 1994

DNA alkylated with [$^3$H]methylnitrosourea (MNU, specific acitivity 18–29 Ci mmol$^{-1}$, Amersham) and treated by heat to remove *N*-alkylated purines (Karran *et al.*, 1979).

*Thymidine kinase (TK) assay*

The TK activities of cell extracts were measured by their ability to phosphorylate thymidine, and calculated as picomoles of thymidine phosphorylated per 10 min per microgram of extract protein (Karran *et al.*, 1977).

### Results

MGMT activities were examined in extracts made from surgical biopsies of two different kinds of melanoma metastases: 20 skin and 25 lymph node. A biopsy from a primary tumour was also obtained. At the time of biopsy none of the patients had received chemotherapy. The quality of extracts made from tumour biopsies was examined by SDS–polyacrylamide gel electrophoresis and by measurements of an independent enzyme hypoxanthine–guanine phosphoribosyl transferase (HGPRT). Extracts showed no large variations using these two parameters (data not shown).

There was a considerable variation in MGMT activity among the tumours (Figure 1). Only lymph node metastases showed MGMT activities above 0.6 pmol mg$^{-1}$ protein, but the average MGMT activities in skin (0.21 ± 0.11 pmol mg$^{-1}$ protein, mean ± s.d.) and lymph node metastases (0.27 ± 0.22 pmol mg$^{-1}$ protein) were similar. The variance, however, was significantly higher ($P < 0.01$) in lymph node than in skin metastases.

It is possible to study how the MGMT activity differs between separate metastases in the same individual, since biopsies from two or more metastases were available from seven of the patients (Figure 2). In three of the seven subjects the difference in MGMT activity between metastases was more than 2-fold.

Lee *et al.* (1992) have shown that the levels of MGMT protein analysed with polyclonal antibodies varies within melanoma metastases, and that only some of the cells in the tumours express the MGMT protein. We also analysed the MGMT activities in different parts of individual metastases (Figure 3). A heterogeneity in MGMT activity within individual metastases was registered but the variation was not as pronounced as that between different metastases (Figure 3).

**Figure 1** The distribution of MGMT activities in crude cell extracts from 25 lymph node (O) and 20 skin metastases (●).

In only two of ten metastases a more than 2-fold difference was observed in different parts of the tumour.

The MGMT expression in some cultured cell lines has been shown to be co-regulated with the expression of two unrelated enzymes, galactokinase and thymidine kinase (TK) (Karran *et al.*, 1990). The mechanisms causing this phenomenon are unknown. To find out if co-regulation also occurs *in vivo*, we analysed TK activities in several extracts, but found no correlation between the MGMT and TK activities in melanoma metastases ($r = 0.26$).

### Discussion

Approximately 20% of tumour cell lines exhibit the mex$^-$ phenotype (Day *et al.*, 1980*a*, *b*; Sklar and Strauss, 1981; Yarosh *et al.*, 1983). It is of importance to find out if this is an *in vitro* artifact, or if it reflects the situation in the tumours of patients *in vivo*. We therefore examined the MGMT activity in biopsies of melanoma metastases, and observed that low levels of MGMT activity (mex$^-$; MGMT activity ⩽0.05 pmol mg$^{-1}$ protein) were rarer in these metastases than in tumour cell lines. The explanation for this could be that during establishment of tumour cell lines mex$^-$ cells might have a growth advantage.

Our results show similar mean MGMT activities in lymph node and skin metastases (Figure 1). Interestingly, the variance among the lymph node metastases was significantly higher than among the skin metastases. In evaluating these results we must take into consideration the fact that the tumours contain normal stroma and blood cells in addition to tumour cells. The results represent the average MGMT activity of all cells in the biopsy, not just tumour cells. It is thus possible that detectable MGMT activities of some tumours depend on non-tumour cells while the melanoma cells may be mex$^-$.

**Figure 2** MGMT activities in seven patients from whom two or more samples were obtained (O, lymph node metastases; ●, skin metastases; Δ, primary tumour).

**Figure 3** MGMT activities in extracts from two separate parts of the same tumour in ten metastases (symbols as in Figure 1).

Heterogeneity in MGMT activity between different metastases in a patient seems to be relatively frequent. This result is consistent with the possibility that primary tumours may contain several subpopulations of tumour cells with metastatic properties which differ in MGMT activity. The metastatic process could then result in the dominance of different tumour cell populations in different metastases. Alternatively, the cells in the primary tumour may have a uniform activity of MGMT, and this original cell population could also be present in some of the metastases, while in other metastases subpopulations of cells with different MGMT activities may arise during tumour progression. If such cells have a growth advantage over the original cell population they could become the dominating cells in the metastasis.

In the present study we have found a wide variation in MGMT activity between melanoma metastases. We now plan to investigate whether MGMT activity is of importance for resistance to clinical chemotherapy containing DTIC in malignant melanoma.

**Acknowledgements**
This investigation was supported by the Stockholm Cancer Society, King Gustav V's Jubilee Fund, the Swedish Cancer Society and the Thure Carlsson Fund. We thank Dr P Karran for supplying *M. luteus* DNA containing [$^3$H]$O^6$-methylguanine residues.

**References**

BRADFORD MM. (1976). A rapid and sensitive method for the quantitation of microgram quantities of protein utilizing the principle of protein–dye binding. *Ann. Biochem.*, **72**, 248–254.

CAO E-H, FAN X-A, YUAN X-H, XIN S-M, LIU Y-Y AND YU H-T. (1991). Levels of $O^6$-methylguanine acceptor protein in extracts of human breast tumor tissues. *Cancer Biochem. Biophys.*, **12**, 53–58.

CITRON M, DECKER R, CHEN S, SCHNEIDER S, GRAVER M, KLEYNERMAN L, KAHN LB, WHITE A, SCHOENHAUS M AND YAROSH D. (1991). $O^6$-methylguanine–DNA methyltransferase in human normal and tumor tissue from brain, lung and ovary. *Cancer Res.*, **51**, 4131–4134.

COMIS RL. (1976). DTIC (NSC 45388) in malignant melanoma. *Cancer Treat. Rep.*, **60**, 165–176.

DAY III RS, ZIOLKOWSKI CH, SCUDIERO DA, MEYER SA AND MATTERN MR. (1980*a*). Human tumor cell strains defective in the repair of alkylation damage. *Carcinogenesis*, **1**, 21–32.

DAY III RS, ZIOLKOWSKI CHJ, SCUDIERO DA, MEYER SA, LUBINIECKI AS, GIARDI AJ, GALLOWAY SM AND BYNUM GD. (1980*b*). Defective repair of alkylated DNA by human tumor and SV40-transformed human cell strains. *Nature*, **288**, 724–727.

EGYHÁZI S, BERGH J, HANSSON J, KARRAN P AND RINGBORG U. (1991). Carmustine-induced toxicity, DNA crosslinking and $O^6$-methylguanine–DNA methyltransferase activity in two human lung cancer cell lines. *Eur. J. Cancer*, **27**, 1658–1662.

ERICKSON LC, BRADLEY MO, DUCORE JM, EWIG RAG AND KOHN KW. (1980*a*). DNA crosslinking and cytotoxicity in normal and transformed human cells treated with antitumor nitrosoureas. *Proc. Natl Acad. Sci. USA*, **77**, 467–471.

ERICKSON LC, LAURENT G, SHARKEY NA AND KOHN KW. (1980*b*). DNA cross-linking and monoadduct repair in nitrosourea-treated human tumor cells. *Nature*, **288**, 727–729.

FERGUSON RJ, ANDERSON LE, MACPHERSON JS, ROBINS P AND SMYTH JF. (1988). Activity of a new nitrosourea (TCNU) in human lung cancer xenografts. *Br. J. Cancer*, **57**, 339–342.

FROSINA G, ROSSI O, ARENA G, GENTILE SL, BRUZZONE E AND ABBONDANDOLO A. (1990). $O^6$-alkylguanine-DNA alkyltransferase activity in human brain tumors. *Cancer Lett.*, **55**, 153–158.

HOUGHTON AN, LEGHA S AND BAJORIN DF. (1992). Chemotherapy for metastatic melanoma. In *Cutaneous Melanoma*, Balch CM, Houghton AN, Milton GW, Sober AJ and Soong S (eds) pp. 498–508. J.B. Lippincott: Philadelphia.

KARRAN P, MOSCONA A AND STRAUSS B. (1977). Developmental decline in DNA repair in neural retina cells of chick embryos. Persistent deficiency of repair competence in a cell line derived from late embryos. *J. Cell. Biol.*, **74**, 274–286.

KARRAN P, LINDAHL T AND GRIFFIN B. (1979). Adaptive response to alkylating agents involves alteration in situ of $O^6$-methylguanine residues in DNA. *Nature*, **280**, 76–77.

KARRAN P, STEPHENSON C, MACPHERSON P, CAIRNS-SMITH S AND PRIESTLY A. (1990). Coregulation of the human $O^6$-methylguanine-DNA methyltransferase with two unrelated genes that are closely linked. *Cancer Res.*, **50**, 1532–1537.

LEE SM, RAFFERTY JA, ELDER RH, FAN C-Y, BROMLEY M, HARRIS M, THATCHER N, POTTER PM, ALTERMATT HJ, PERINAT-FREY T, CERNY T, O'CONNOR PJ AND MARGISON GP. (1992). Immunohistological examination of the inter- and intracellular distribution of $O^6$-alkylguanine DNA-alkyltransferase in human liver and melanoma. *Br. J. Cancer*, **66**, 355–360.

MEER L, JANZER RC, KLEIHUES P AND KOLAR GF. (1986). In vivo metabolism and reaction with DNA of the cytostatic agent, 5-(3,3-dimethyl-1-triazeno)imidazole-4-carboxamide. *Biochem. Pharmacol.*, **35**, 3243–3247.

MINEURA K, IZUMI I, KUWAHARA N AND KOWADA M. (1994). $O^6$-methylguanine-DNA methyltransferase activity in cerebral gliomas. A guidance for nitrosourea treatment? *Acta Oncol.*, **33**, 29–32.

MYRNES B, GIERCKSKY K AND KROKAN H. (1983). Interindividual variation in the activity of $O^6$-methylguanine–DNA methyltransferase and uracil-DNA glycosylase in human organs. *Carcinogenesis*, **4**, 1565–1568.

MYRNES B, NORSTRAND K, GIERCKSKY K, SJUNNESKOG C AND KROKAN H. (1984). A simplified assay for $O^6$-methylguanine-DNA methyltransferase activity and its application to human neoplastic and non-neoplastic tissues. *Carcinogenesis*, **5**, 1061–1064.

PEGG AE. (1990). Mammalian $O^6$-alkylguanine-DNA alkyltransferase: regulation and importance in response to alkylating carcinogenic and therapeutic agents. *Cancer Res.*, **50**, 6119–6129.

RINGBORG U, RUDENSTAM C-M, HANSSON J, HAFSTRÖM L, STENSTAM B AND STRANDER H. (1989). Dacarbazine versus dacarbazine-vindesine in disseminated malignant melanoma: a randomized phase II study. *Med. Oncol. Tumor Pharmacother.*, **6**, 285–289.

RINGBORG U, JUNGNELIUS U, HANSSON J AND STRANDER H. (1990). Dacarbazine-vindesine-cisplatin in disseminated malignant melanoma: a phase I–II trial. *Am. J. Clin. Oncol. (CCT)*, **13**, 214–217.

SCUDIERO DA, MEYER SA, CLATTERBUCK BE, MATTERN MR, ZIOLKOWSKI CHJ AND DAY III RS. (1984). Sensitivity of human cell strains having different abilities to repair $O^6$-methylguanine in DNA to inactivation by alkylating agents including chloroethylnitrosoureas. *Cancer Res.*, **44**, 2467–2474.

SKLAR R AND STRAUSS B. (1981). Removal of $O^6$-methylguanine from DNA of normal and xeroderma pigmentosum-derived lymphoblastoid cells. *Nature*, **289**, 417–420.

WIESTLER O, KLEIHUES P AND PEGG AE. (1984). $O^6$-alkylguanine-DNA alkyltransferase activity in human brain and brain tumors. *Carcinogenesis*, **5**, 121–124.

YAROSH DB, FOOTE RS, MITRA S AND DAY III RS. (1983). Repair of $O^6$-methylguanine in DNA by demethylation is lacking in mer$^-$ human tumor cell strains. *Carcinogenesis*, **4**, 199–205.